# Supplementary material for: A Global Screen for Assembly State Changes of the Mitotic Proteome by SEC-SWATH-MS
Source: Cell Syst. 2020 Feb 26;10(2):133–155.e6. doi: 10.1016/j.cels.2020.01.001 (PMC7042714; doi:10.1016/j.cels.2020.01.001)

Q9Y5J5 | PHLA3_HUMAN | PHLDA3 TIH1
Monomer MW [kDa]: 13.891 Monomer expected elution fraction: 57

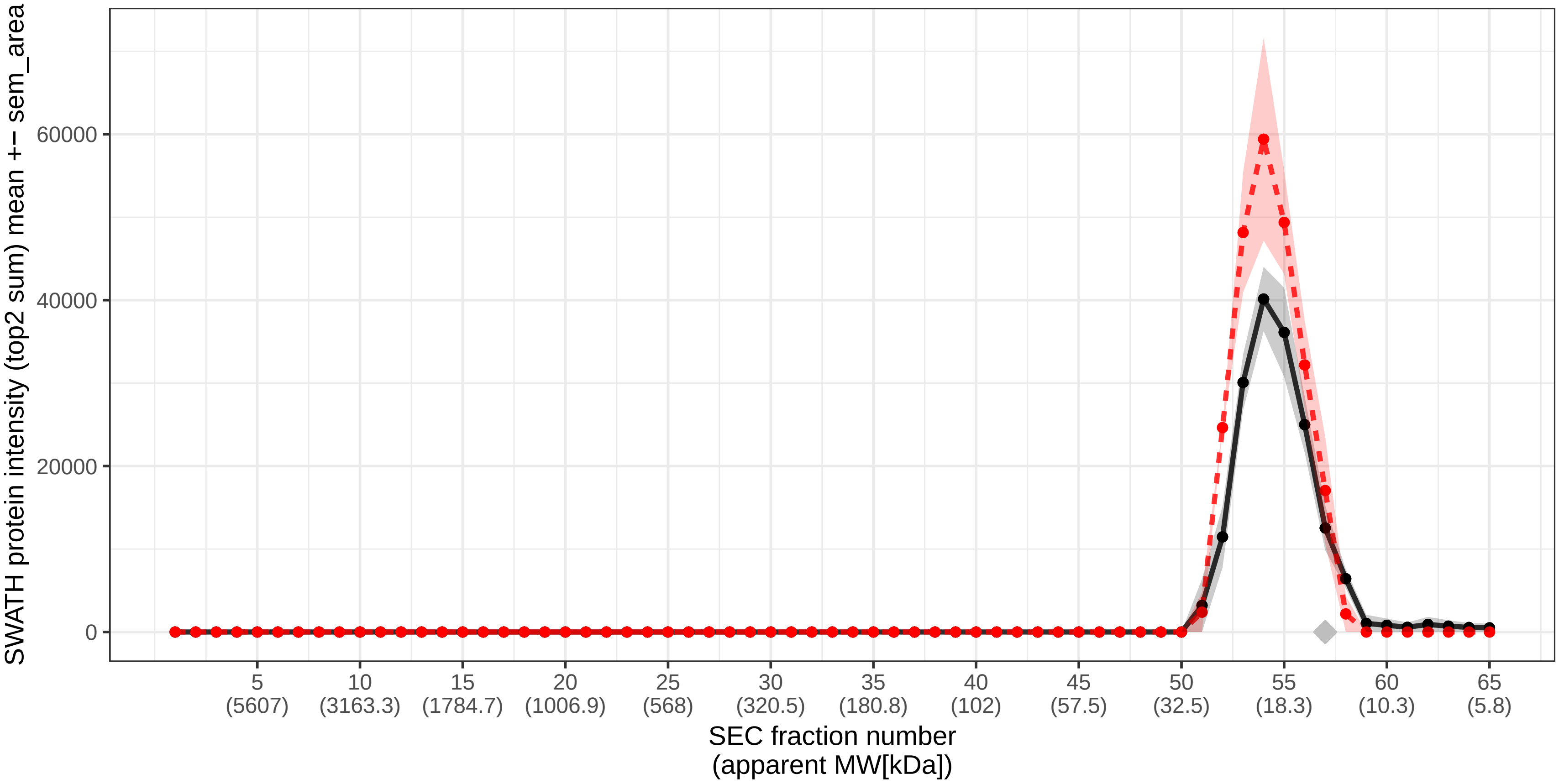

Supplement: Data S1. SEC-SWATH-MS Protein Chromatograms, Related to Figure 1 [file mmc6.zip › SECchrom_Q9Y5J5_PHLA3_HUMAN_PHLDA3_TIH1.pdf]
